# Dark Personality Traits and Burnout as Predictors of Relational Aggression: A Study in Young Adults

**DOI:** 10.7759/cureus.89758

**Published:** 2025-08-10

**Authors:** Simran Sandhu, Ashvin Chouhan, Akanksha Singh, Vijay Niranjan, Medha Pandey

**Affiliations:** 1 Psychiatry, Mahatma Gandhi Memorial Medical College, Indore, IND; 2 Psychiatry and Behavioral Sciences, Mahatma Gandhi Medical College and Research Institute, Shahdol, IND

**Keywords:** burnout, dark triad personality traits, relational aggression, substance use, young adults

## Abstract

Background

Relational aggression - covert behaviors such as social exclusion, guilt induction, and malicious humor - is increasingly observed among young adults. While dark personality traits (narcissism, Machiavellianism, and psychopathy) are known to predict such behaviors, the influence of burnout - a state of emotional exhaustion and detachment - on this relationship remains underexplored.

Aim

This study aimed to examine how dark triad traits and burnout interact in predicting relational aggression in a non-clinical young adult population.

Methods

A total of 200 participants aged 18-30 years completed standardized self-report measures assessing dark triad traits, burnout, and relational aggression. Sociodemographic variables, including gender and substance use, were also analyzed.

Results

Higher levels of dark traits were associated with increased relational aggression. Males scored significantly higher in psychopathy and indirect aggression subtypes. Participants reporting cannabis and alcohol use showed higher aggression, with cannabis users scoring the highest. Those with high dark traits but low burnout reported the most aggression, while high burnout weakened the expression of these traits. This suggests burnout may temporarily dampen socially harmful behaviors.

Conclusion

Dark personality traits contribute to indirect aggression, but this effect is influenced by emotional exhaustion. Burnout may act as a psychological buffer, reducing the behavioral expression of these traits. These findings highlight the importance of assessing both personality and emotional state in understanding interpersonal difficulties, even in non-clinical populations.

## Introduction

The increasing prevalence of stress, emotional exhaustion, and deteriorating interpersonal functioning among young adults has become a growing psychological concern. This stage of life often brings many challenges, such as academic pressure, career uncertainty, changing relationships, and identity struggles.

For many young adults, these stressors accumulate, leading to burnout - a syndrome traditionally studied in occupational settings but now recognized as a pervasive response to chronic stress across diverse domains [1,2]. Burnout is characterized by emotional exhaustion, depersonalization, and a reduced sense of personal accomplishment [3]. It impairs interpersonal functioning and increases vulnerability to maladaptive behaviors [2].

Emerging research suggests that certain personality traits may predispose individuals to maladaptive responses under stress [4,5]. Of particular interest are the dark triad traits (narcissism, Machiavellianism, and psychopathy) that, while distinct, share common features such as emotional coldness, manipulation, and self-centeredness [4]. Narcissism is characterized by grandiosity, a heightened sense of entitlement, and a fragile self-esteem that is highly sensitive to criticism. Under stress, narcissistic individuals may cope maladaptively by externalizing blame, engaging in defensive hostility, or seeking validation through manipulative means [5]. Machiavellianism involves a strategic, manipulative interpersonal style, marked by cynicism, deceit, and a focus on personal gain. When stressed, Machiavellian individuals are more likely to resort to calculated social manipulation or exploitative behavior rather than emotional expression or help-seeking [6]. Psychopathy, particularly in its subclinical form, is defined by impulsivity, callousness, and a lack of empathy or remorse. Under stress, individuals high in psychopathy may display aggressive or risk-taking behavior, showing little concern for social norms or the well-being of others [7]. Given their interpersonal nature, these traits are often expressed not through physical aggression but through relational aggression.

Relational aggression is a form of non-physical, indirect aggression that aims to harm others by damaging or manipulating their social relationships, reputation, or feelings of inclusion. Unlike overt aggression, which involves direct verbal or physical hostility, relational aggression is subtle, often hidden behind socially acceptable behavior, and typically occurs within close relationships, peer groups, or social networks [8].

Relational aggression commonly manifests in three key forms: social exclusion, guilt induction, and malicious humor. Social exclusion involves deliberately isolating someone from a group, event, or social activity to punish, control, or diminish their social standing. Guilt induction, on the other hand, manipulates individuals by making them feel responsible for another person’s distress or unhappiness. Malicious humor refers to the use of sarcasm, ridicule, or seemingly harmless “jokes” to belittle, embarrass, or undermine someone, often under the guise of humor, while inflicting psychological harm.

Prevalence rates of relational aggression vary across settings, with studies reporting that approximately 17% to 25% of adolescents and young adults engage in relationally aggressive behaviors, and workplace studies indicating rates as high as 28% [9]. It often serves psychological functions such as asserting dominance, managing insecurities, or retaliating against perceived threats or slights [9]. Unlike physical aggression, relational aggression can be difficult to identify, as it is often cloaked in humor, passive behavior, or strategic manipulation. Notably, individuals high in dark traits may be especially likely to use relational aggression as a socially acceptable form of manipulation, especially in high-pressure environments.

While traits such as narcissism, Machiavellianism, and psychopathy have been linked to aggression under stress, it is important to distinguish stress from burnout. Stress involves acute pressure, whereas burnout reflects prolonged emotional exhaustion, detachment, and reduced functioning. Though traditionally studied in occupational contexts, burnout has also been associated with irritability, emotional dysregulation, and, in some cases, aggression [10]. However, findings are mixed; some studies suggest burnout may suppress aggression via withdrawal, while others indicate it lowers impulse control, increasing hostility [11,12].

This study adopts a trait-state interaction perspective, where stable personality traits interact with transient emotional states to influence behavior. According to the trait activation theory, certain traits manifest behaviorally only when relevant situational cues activate them [13]. Likewise, the diathesis-stress model posits that trait vulnerabilities interact with emotional stressors (e.g., burnout) to produce maladaptive outcomes [14].

Despite these theoretical frameworks, the joint role of burnout and dark traits in predicting relational aggression remains underexplored, particularly in non-clinical young adult populations in India. Investigating how trait dispositions and emotional exhaustion interact may enhance the understanding of covert maladaptive behaviors and inform culturally relevant mental health interventions.

The aim of this study is to examine the relationship between dark triad personality traits, burnout, and relational aggression among young adults.

## Materials and methods

This study employed a cross-sectional, observational research design to examine the relationship between dark triad personality traits, burnout, and relational aggression in young adults. Participants were recruited through convenience sampling via online platforms, including social media networks, email, and messaging groups.

Inclusion criteria

The study included individuals aged 18 to 30 years, able to understand English, and willing to provide informed consent.

Exclusion criteria

The exclusion criteria were as follows: a history of major neuropsychiatric illness, incomplete or inconsistent questionnaire responses, and withdrawal of consent at any point during data collection.

Ethical consideration

Ethical approval was obtained from the Institutional Ethics Committee prior to data collection. All participants were informed of the study’s purpose, procedures, and their rights as research subjects. Participation was entirely voluntary, and responses were collected anonymously to maintain confidentiality and reduce social desirability bias.

Data collection

Data collection was carried out using a self-administered online survey comprising demographic information and standardized psychological instruments. The Short Dark Triad (SD3) scale (α = 0.80) [15] was used to measure narcissism, Machiavellianism, and psychopathy. This 27-item measure includes nine items for each trait, rated on a Likert scale. Higher scores indicated greater expression of the respective traits. Burnout was assessed using the Oldenburg Burnout Inventory (OLBI) (α = 0.83) [16], a 16-item tool measuring two dimensions: exhaustion and disengagement. Participants responded using a 4-point Likert scale, with higher scores reflecting greater burnout. Relational aggression was measured using the Indirect Aggression Scale-Aggressor Version (α = 0.87), developed to assess subtle forms of interpersonal aggression, including social exclusion, guilt induction, and malicious humor [17]. The 25-item scale uses a 5-point Likert format, with higher scores reflecting more frequent engagement in relationally aggressive behaviors.

Sample size

The sample size was determined a priori based on power analysis for both correlation and multiple regression. For correlation, assuming a medium effect size (r = 0.30), α = 0.05, and power = 0.80, the required sample size was approximately 85 participants. For multiple regression, applying Cohen’s rule of thumb (N ≥ 50 + 8k predictors) for the planned number of predictors indicated a requirement of about 200 participants. To meet the larger of these requirements and to allow for possible exclusions, a target sample size of 200 was set.

Statistical analysis

**Figure 1:**
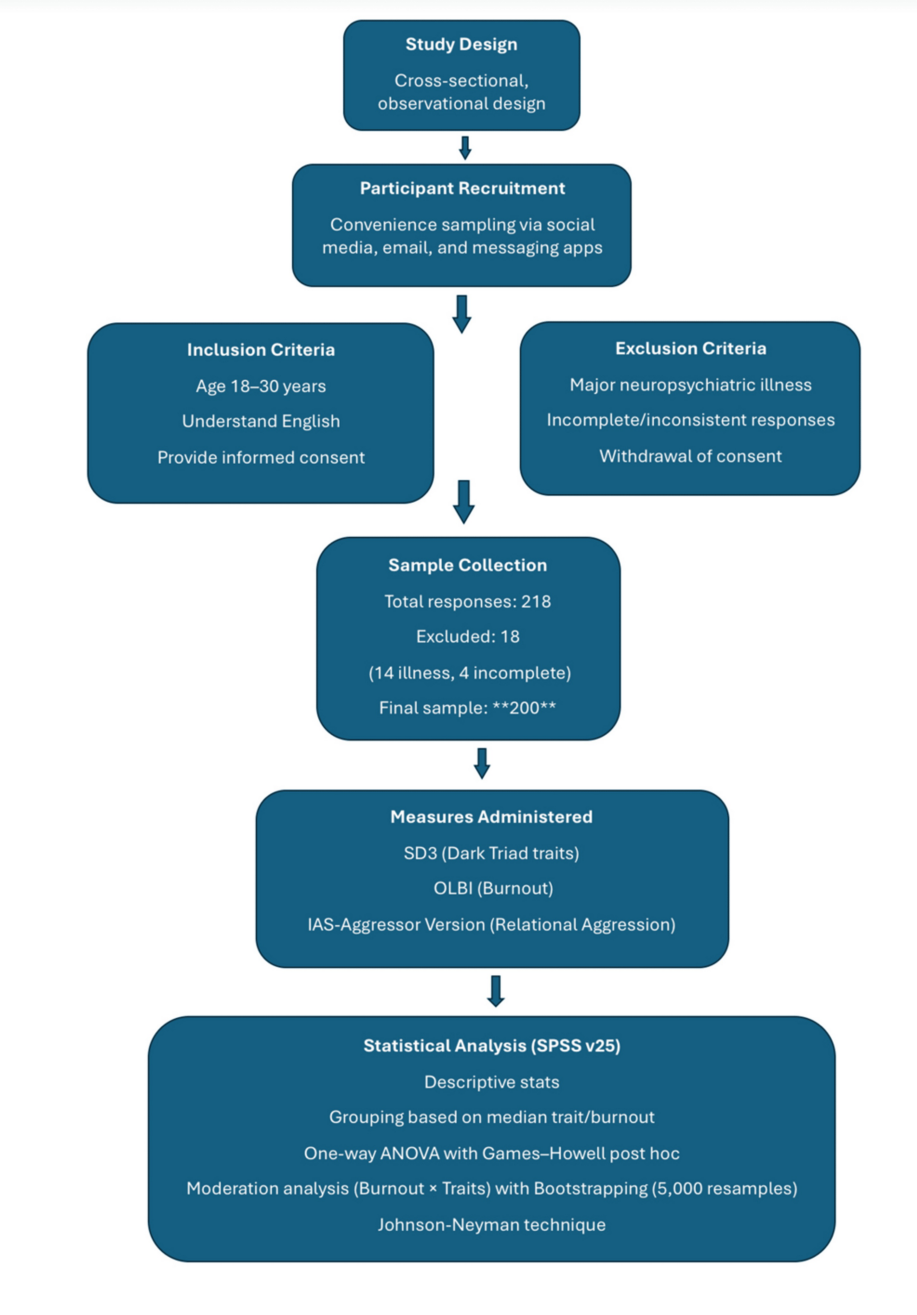
Study design and methodology overview SD3, Short Dark Triad; OLBI, Oldenburg Burnout Inventory; IAS, Indirect Aggression Scale; ANOVA, analysis of variance

Statistical analysis was conducted using SPSS Version 25 (IBM Corp., Armonk, NY). Descriptive statistics were first computed for all sociodemographic variables, including frequencies and percentages for categorical data such as age, sex, education, and income. Mean, standard deviation, and median scores were then calculated for each psychological scale: dark traits, burnout, and aggression. Based on the median values of total dark traits and total burnout, participants were divided into four groups: Low trait-low burnout, low trait-high burnout, high trait-low burnout, and high trait-high burnout. Aggression scores across these groups were compared using one-way ANOVA. Levene’s test was applied to assess homogeneity of variances, and due to violation of this assumption, Games-Howell post hoc tests were used. To examine whether burnout moderated the relationship between dark traits and aggression, a moderation analysis was conducted using multiple linear regression with centered predictors and an interaction term. This was followed by bootstrapping with 5,000 resamples to derive robust confidence intervals for the interaction. To further explore the nature of the moderation, the Johnson-Neyman technique was applied to identify the specific range of burnout scores where the relationship between dark traits and aggression remained statistically significant. Statistical significance was set at p < 0.05 (Figure 1).

## Results

A total of 218 individuals responded to the online survey. Of these, 14 participants were excluded due to a self-reported history of neuropsychiatric illness, and an additional 4 participants were excluded for submitting incomplete responses. After applying these exclusion criteria, the final sample size for analysis was 200 participants. The study revealed that higher levels of dark triad traits were significantly associated with increased relational aggression. The mean total dark trait score was 78.79 (SD = 11.22), total burnout was 39.97 (SD = 5.60), and total aggression was 43.90 (SD = 15.86) (Table 1). Among subgroups, males scored significantly higher than females on psychopathy and on relational aggression subtypes, including guilt induction, malicious humor, and social exclusion. Participants with a history of substance use (11%), particularly cannabis users (mean aggression = 58.80) and alcohol users (mean = 51.73), had significantly higher aggression scores compared to non-users (p = 0.042) (Appendices).

**Table 1:** Descriptive statistics for total psychological scale scores

Variable	Median	Mean	SD	Min	Max
Total dark traits	79.0	78.79	11.22	47	109
Total burnout	39.5	39.97	5.60	21	60
Total aggression	40.0	43.90	15.86	25	92

Comparing trait-burnout combinations, those with high dark traits and low burnout exhibited the highest aggression (mean = 49.05), followed by the high trait-high burnout group (mean = 47.05) and low trait-high burnout group (mean = 43.13), with the lowest aggression observed in the low trait-low burnout group (mean = 37.53) (Table 2). One-way ANOVA confirmed that these differences were statistically significant (F(3, 196) = 6.42, p < 0.001; Table 2), and post hoc Games-Howell tests showed that aggression in the high trait-low burnout group was significantly higher than that in both low trait groups (p < 0.001 and p = 0.016, respectively) (Table 3).

**Table 2:** Comparison of total aggression scores across trait and burnout groups

Group	N (%)	Mean difference in aggression	SD	Min	Max	F	P-value
Low trait, low burnout	62 (31)	37.53	12.82	25	76	6.42	<0.01
Low trait, high burnout	40 (20)	43.13	14.81	26	92		
High trait, low burnout	58 (29)	49.05	16.81	25	86		
High trait, high burnout	40 (20)	47.05	16.71	25	77		

**Table 3:** Post hoc (Games-Howell) comparisons of aggression scores across trait-burnout groups

Comparison	Mean difference in aggression scores	SE	P-value
Low trait, low burnout vs low trait, high burnout	-5.59	2.85	0.212
Low trait, low burnout vs high trait, low burnout	-11.52	2.74	0.001
Low trait, low burnout vs high trait, high burnout	-9.52	3.10	0.016
Low trait, high burnout vs high trait, low burnout	-5.93	3.22	0.261
Low trait, high burnout vs high trait, high burnout	-2.00	3.44	0.937
High trait, low burnout vs high trait, high burnout	3.93	3.53	0.683

**Figure 2:**
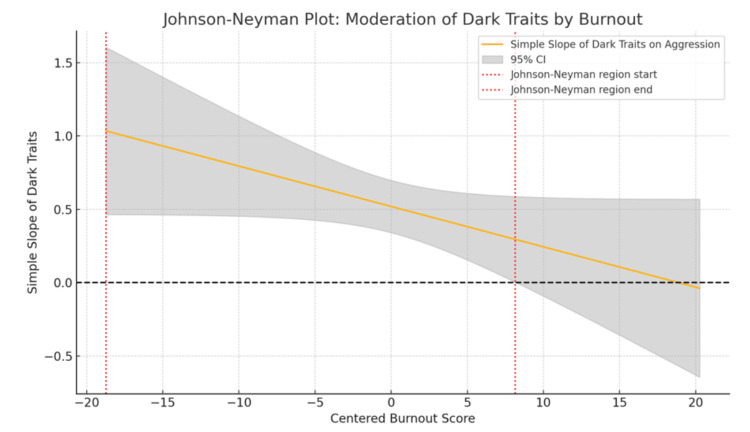
Johnson-Neyman plot showing moderation of the relationship between dark traits and aggression by burnout This figure illustrates the Johnson-Neyman analysis, which identifies the range of burnout scores at which the relationship between dark traits and aggression is statistically significant. The x-axis represents centered burnout levels, while the y-axis shows the strength (slope) of the association between dark traits and aggression. The solid blue line indicates how this relationship changes across burnout levels. The shaded grey region represents the 95% confidence interval around the slope. The two vertical red dotted lines mark the Johnson-Neyman bounds - the range within which the relationship is statistically significant. Specifically, dark traits significantly predict aggression at lower to moderate levels of burnout, but this association becomes non-significant at higher burnout levels.

Moderation analysis showed that while dark traits significantly predicted aggression (β = 0.519, p < 0.001), burnout alone did not (p = 0.835). The interaction term (dark traits × burnout) had a negative coefficient (β = -0.028, p = 0.061), and bootstrapping confirmed that the interaction was significant, with a 95% confidence interval of (-0.059, -0.007), suggesting that burnout weakens the link between dark traits and aggression (Table 4). The Johnson-Neyman technique further showed that this relationship was statistically significant only at low to moderate levels of burnout (up to +0.4 SD), becoming non-significant at higher levels, highlighting burnout’s potential role as a temporary buffer against the expression of socially aversive traits (Figure 2).

**Table 4:** Moderation analysis with bootstrapped confidence intervals

Predictor	Coefficient	Std. error	95% CI lower	95% CI upper	P-value	Bootstrapped, 95% CI
Dark traits	0.519	0.091	0.340	0.698	0.000	-
Burnout	0.038	0.180	-0.318	0.393	0.835	-
Interaction (dark traits x burnout)	-0.028	0.015	-0.056	0.001	0.061	-0.059, -0.007

## Discussion

This study investigated the association between dark personality traits, burnout, and indirect aggression in a non-clinical adult population. Specifically, it aimed to explore not only the direct relationship between dark traits and relational aggression but also the moderating role of burnout in this association.

The sample comprised predominantly young adults with an approximately equal gender distribution (51% male, 48% female). A large proportion were single (83%) and highly educated, with 50% being graduates and 32% having completed postgraduate education. In terms of occupational status, 32.5% were unemployed, while 31.5% were employed in professional roles. Over half of the sample (57%) reported a monthly income of ₹1,00,000 or more, placing them in a higher income bracket (Appendices A-C).

Findings indicated a moderate presence of socially aversive personality traits within this non-clinical sample. Among the dark triad components, narcissism had the highest mean score (28.64, SD = 5.49), followed by Machiavellianism (27.27, SD = 4.62) and psychopathy (22.71, SD = 5.50). This pattern aligns with existing literature, which consistently reports that narcissistic and Machiavellian traits are more prevalent than psychopathy in general populations, given the latter’s association with overt antisocial behavior [4,18] (Table 1).

The average burnout score was 39.68 (SD = 7.89), with mean subscale scores of 19.83 (SD = 4.44) for exhaustion and 19.85 (SD = 4.45) for disengagement. These values reflect moderate levels of burnout, consistent with previous studies conducted among young adults and students in demanding environments, where prolonged psychological stress and emotional fatigue are increasingly reported [19,20]. Relational aggression scores averaged 43.35 (SD = 11.76), with higher contributions from the domains of malicious humor (15.64, SD = 5.71) and social exclusion (17.06, SD = 5.98), while guilt induction was comparatively lower (10.66, SD = 4.22). These findings resonate with prior research suggesting that among high-functioning populations, such as educated young adults, aggression is more likely to manifest in covert, socially manipulative forms rather than through overt physical or verbal means [21] (Table 1).

Subgroup analyses indicated that male participants scored significantly higher than females on psychopathy, as well as on all three dimensions of indirect aggression-guilt induction, malicious humor, and social exclusion. This is congruent with literature documenting gender-based differences, where males tend to display greater levels of antagonism, emotional detachment, and manipulativeness - traits characteristic of psychopathy and Machiavellianism [22,23].

Regarding substance use, 11% of participants reported a history of use, primarily involving alcohol and cannabis. These individuals demonstrated significantly higher levels of aggression compared to non-users (p = 0.042). Among them, cannabis users reported the highest mean aggression score (M = 58.80), followed by alcohol users (M = 51.73), while tobacco users exhibited the lowest aggression levels (M = 32.50), despite experiencing higher levels of burnout. However, comparisons between individual substance groups and non-users did not reach statistical significance, possibly due to limited subgroup sizes. These findings are consistent with prior research linking cannabis use with increased impulsivity, irritability, and emotional dysregulation [24].

Group comparisons based on combinations of dark traits and burnout levels revealed that individuals high in dark traits exhibited greater indirect aggression, particularly when emotional depletion was low. Conversely, those in the low trait-high burnout group had the lowest aggression scores, suggesting that burnout, in the absence of pronounced trait vulnerability, may dampen interpersonal reactivity (Table 2). Post hoc analyses (Games-Howell) confirmed significantly higher aggression in the high trait-low burnout group compared to both low trait groups (p < 0.05). Among individuals with high dark traits, those with high burnout demonstrated slightly lower aggression, although the difference was not always statistically significant (Table 3).

To further investigate the conditional nature of this relationship, a moderation analysis was conducted with burnout as a moderator. Regression analysis indicated that while dark traits significantly predicted aggression, burnout alone did not. However, the interaction term (dark traits × burnout) approached significance, suggesting a potential moderating role. Bootstrapping with 5,000 resamples confirmed the robustness of this interaction, lending support to the hypothesis that burnout moderates the relationship between dark traits and aggression. Specifically, as burnout levels increased, the association between dark traits and aggression weakened, corroborating earlier group-based findings (Table 4).

To provide further clarity, the Johnson-Neyman technique was employed. This analysis demonstrated that the association between dark traits and aggression was statistically significant only at low to moderate levels of burnout (up to approximately +0.4 SD above the mean) and became non-significant at higher burnout levels (Figure 2). This finding supports the conceptualization of burnout not merely as a state of emotional exhaustion but also as a potential inhibitory mechanism against the enactment of socially harmful behaviors. Although Indian research has primarily focused on burnout in academic and occupational contexts [19], emerging studies have begun to explore its emotional blunting effects [25]. Scholars such as Bianchi et al. [10] and Schaufeli and Taris [11] have described burnout as a state of emotional depletion, which may limit an individual’s capacity to engage in manipulative or aggressive behaviors, even among those with elevated dark trait profiles. These findings thus lend support to integrated trait-state behavioral models. In clinical psychiatry, they highlight the importance of assessing both dispositional traits and current emotional states such as burnout during diagnostic evaluations, particularly in individuals who appear well-functioning yet present with persistent interpersonal difficulties. Such comprehensive assessment approaches may enhance risk evaluation, inform treatment planning, and promote early intervention in personality-related behavioral disturbances.

The strong association observed between dark personality traits and relational aggression underlines the relevance of personality screening in clinical settings, especially among young adults who may not present with formal psychopathology but experience chronic interpersonal dysfunction. These personality traits are often overlooked in standard assessments, yet existing evidence suggests that they can predict poor therapeutic alliance, treatment non-adherence, and relational conflict [26].

Furthermore, the current findings suggest that emotional exhaustion may temporarily suppress the behavioral expression of dark traits. However, as burnout resolves, the risk of resurgent aggressive behaviors may increase. This underscores the importance of multifaceted clinical interventions aimed at not only reducing burnout but also addressing the underlying personality dynamics that contribute to maladaptive interpersonal patterns. Therapeutic modalities such as dialectical behavior therapy and schema therapy may prove particularly useful in this context, as they are designed to enhance interpersonal functioning and emotional regulation skills among individuals with personality vulnerabilities [27,28].

This study’s cross-sectional design limits causal interpretations, and reliance on self-report measures may introduce bias. Although individuals with major neuropsychiatric illness were excluded, some participants reported occasional alcohol or cannabis use, which may act as a potential confounder, particularly for traits such as aggression or psychopathy. Additionally, the sample was predominantly urban, educated, and high-income, limiting generalizability. Detailed substance use patterns were not assessed.

Future research should explore in greater depth the roles of gender and substance use in moderating the link between dark personality traits and relational aggression. Longitudinal studies are essential to examine the dynamic interplay between dark traits and burnout over time and to determine whether recovery from burnout facilitates the reactivation of previously suppressed aggressive behaviors. In particular, clinical intervention-based longitudinal studies are needed to assess how targeted therapeutic strategies can mitigate the expression of relational aggression in individuals exhibiting both high dark traits and burnout.

## Conclusions

This study explored the interplay between socially aversive personality traits (narcissism, Machiavellianism, and psychopathy), emotional exhaustion (burnout), and indirect aggression in a non-clinical sample of young adults. The findings revealed that individuals with higher levels of dark personality traits were more likely to engage in subtle forms of aggression, including social exclusion, malicious humor, and guilt induction. These behaviors were more prevalent among males and individuals reporting occasional use of substances such as alcohol and cannabis. Notably, emotional exhaustion appeared to reduce the expression of these aggressive tendencies, suggesting that burnout may temporarily inhibit maladaptive interpersonal behavior, even in those predisposed to it.

These findings underscore the need for integrated assessments in clinical practice that consider both personality traits and current emotional states, particularly in individuals presenting with interpersonal difficulties. In non-clinical settings such as educational institutions or workplaces, screening for burnout and early signs of maladaptive personality traits could inform the development of supportive policies and targeted interventions to improve relational dynamics, reduce conflict, and enhance overall psychosocial functioning.

## Appendices

Appendix A

**Table 5:** Sociodemographic characteristics of the study sample

Variable	N	%
Age (years)		
21-25	96	48.0
26-30	84	42.0
18-20	20	10.0
Sex		
Male	102	51.0
Female	96	48.0
Prefer not to say	2	1.0
Education		
Graduate	100	50.0
Postgraduate	64	32.0
Intermediate/diploma	20	10.0
High school	16	8.0
Occupation		
Unemployed	65	32.5
Professional	63	31.5
Semi-professional	23	11.5
Clerical	20	10.0
Skilled	11	5.5
Unskilled	9	4.5
Semi-skilled	9	4.5
Marital status		
Single	166	83.0
Married	28	14.0
Other	6	3.0
Income (INR)		
Upper+ (≥1,00,000)	114	57.0
Middle (30,000–49,999)	23	11.5
Upper-middle (50,000–74,999)	22	11.0
Lower (<20,000)	16	8.0
Upper (75,000–99,999)	13	6.5
Lower-middle (20,000–29,999)	12	6.0

Appendix B 

**Table 6:** Gender-based comparison of aggression, burnout, and dark personality traits in young adults

Component	Male (mean)	Female (mean)	T-value	P-value
Total aggression	47.77	39.04	4.13	0.00
Guilt induction	11.68	9.82	3.3	0.001
Malicious humor	17.51	13.69	4.56	0.00
Social exclusion	18.59	15.53	3.23	0.001
Total burnout	39.24	40.2	-1.19	0.237
Exhaustion	19.65	20.22	-1.28	0.201
Disengagement	19.59	19.98	-0.87	0.384
Total dark traits	81.01	76.14	3.13	0.002
Narcissism	29.02	28.08	1.17	0.243
Machiavellianism	27.5	27.22	0.47	0.641
Psychopathy	24.49	20.83	5.23	0.00

Appendix C 

**Table 7:** Mean aggression scores by substance use group

Substance use group	Participants (N)	Mean aggression score	T-value vs. non-users	P-value vs. non-users
All substance users	22	51.59	2.15	0.042
Alcohol	15	51.73	1.84	0.086
Cannabis	5	58.8	1.81	0.143
Tobacco	2	32.5	-2.16	0.253
